# Supplementary material for: Are carbon emissions trading and green financial instruments synergistic? -Comprehensive quantitative research based on content analysis
Source: PLoS One. 2024 Mar 7;19(3):e0298601. doi: 10.1371/journal.pone.0298601 (PMC10919601; doi:10.1371/journal.pone.0298601)
Supplement: S1 Data — (ZIP) [file pone.0298601.s001.zip › Data/地级市Z_F工作报告.zip!/┴╔─■/┴╔╤⌠╩╨/└┤╫╘╣1⁄2╓┌║┼í╛┬φ┐╦╩2╛▌═°í┐_2014.docx]

各位代表：

我代表市人民政府向大会报告工作，请予审议，并请市政协委员和列席会议的同志提出意见。

一、2013年工作回顾

刚刚过去的2013年，是宏观经济环境严峻复杂的一年，也是我们攻坚克难，保持经济平稳增长的一年。

一年来，在省委、省政府和市委的正确领导下，市政府带领全市人民，认真贯彻落实党的十八大精神，推进新型工业化、特色产业化和新型城镇化良性互动发展，圆满完成了市十五届人大一次会议确定的主要任务。地区生产总值增长10%。公共财政预算收入增长12%(按可比口径)。固定资产投资增长20%。城镇居民人均可支配收入增长10%。农村居民人均纯收入增长12%。

(一)新城新市镇建设快速推进，城镇化格局全面拉开

河东新城建设取得新进展。打造生态新城，进行大规模绿化，建设了太子河生态公园、人民公园、北部绿轴等景观工程，提升了新城环境品质。完善北部路网，拓展了新城发展空间。中华大桥维修工程完成。文化中心大剧院主体完工。中南购物中心、红星美凯龙家居广场建设步伐加快。加强新城管理，城市功能日趋完善。培育新城主导产业取得进展，启动了铝合金精深加工产业基地建设，建成起步区路网。庆化公司局部调迁进展顺利。

加强其它新城新市镇基础设施建设，打造整体环境，促进产城联动，推动了“三化”良性互动发展。

(二)工业经济平稳较快增长，产业结构优化升级

以保企业为核心，狠抓经济运行，扶持企业发展。规模以上工业增加值增长11%。

以增量带动结构调整，全年实施重点工业项目131项，其中52个项目建成投产。以淘汰落后产能促使产业转型升级，关闭小钢铁企业67家。以科技创新拉动产业结构优化，高新区被命名为国家知识产权试点园区，忠旺集团获批组建辽宁省重点实验室，高新技术产业快速发展。

园区建设水平大幅提升。芳烃基地跻身中国化工园区20强行列，铝合金精深加工产业基地被列为省级工业园区，经济开发区获批沈阳经济区装备制造业转型升级示范区。六个产业集群全面壮大，推动了工业经济平稳增长。

节能减排扎实推进。建成大气污染治理项目87项，全面完成省下达任务。青山保护、污水治理、淘汰高耗能企业和水泥生产线脱硝改造工作，均走在全省前列。万元地区生产总值综合能耗下降4%，主要污染物实现达标排放。

(三)现代农业长足发展，农业经济发展水平快速提升

出台了一系列政策措施，推动现代农业快速发展。农业综合生产能力稳步提高，现代农业示范区建设扎实推进。粮食总产量达到101万吨，创历史新高。“一县一业”取得新进展。农业机械化水平不断提升，水稻生产基本实现全程机械化。农业产业化步伐加快，建成投资2000万元以上农业产业化项目16个。农产品出口实现新突破。引导土地承包经营权向家庭农场、专业大户流转，促进了农村新型经济组织快速发展。农业科技推广力度加大，高标准建设了现代农林科技示范园。农产品质量安全和监管水平进一步提高。

推进农田基础设施建设，建成高标准农田33万亩。农田水利建设力度加大，防洪灌溉能力显著增强。修复了辽阳县东部山区水毁工程，全面完成灾后重建工作。整治农村环境，村容村貌有较大改观。

(四)现代服务业增势强劲，城区经济发展活力充分释放

集聚区建设取得重大进展。皮装裘皮集聚区晋升为省级示范区，太子河商贸商务区获批省级服务业集聚区，白塔中央商贸区建设步伐加快。海宁皮革城三期、大连友谊商城等项目加速推进，假日水上乐园、香港时代广场、大润发超市正式营业。

特色服务业蓬勃发展。温泉旅游、皮装裘皮购物游持续升温，汤河温泉旅游度假区晋升为国家4A级景区。会展经济日趋活跃，地产名优商品等专业展会已形成品牌效应。

向城区进一步下放32项经济管理权限，扩大了城区发展的自主权，激发了城区经济发展的活力。

(五)城市建设和管理全面加强，人居环境明显改善

老城区改建稳步推进。完成站前广场改建。实施了家乐福地上广场和西关商业步行街绿化景观改造。护城河局部改造工程开工建设。启动了喇嘛园改造工程，违建房屋全部拆除。繁荣路和振兴路两侧改建取得新进展。

城乡基础设施不断完善。市中心客运站投入使用。改扩建城市道路17条。大中修和改扩建县级以上公路248公里、农村公路425公里，建设里程创历史最好水平。建成村级道路270公里。改造城市排水设施，解决了重点区域内涝问题。中心城区供暖备用热源工程基本完工。供应中心城区的天然气输配管线全线贯通。新殡仪服务中心投入使用。加强电力设施建设，电力保障更加有力。

出台了支持房地产和建筑业发展的政策措施，实行项目开工并联审批制度，促进了房地产业健康发展。举办春夏秋三季房交会，为广大市民便捷优惠购房搭建了平台。

城市管理明显加强。开展了侵占居民小区公共场地整治行动，清理工作稳步推进。购置环卫设备，清扫、保洁、除雪机械化水平显著提高。提高了环卫工人工资水平和生活待遇。强化市容、市场和规划监察，市容市貌有了新的改观。

(六)重点领域改革不断深化，招商引资成效显著

深化市属国有企业改革，3户企业实现国有股彻底退出，历史遗留问题逐步解决。企业“退二进三”步伐加快，闲置土地得到盘活。集体企业改革稳步推进。文化体制改革平稳进行，新华书店完成事转企改制。基层医药卫生体制改革不断深化。食品药品监管体制改革有序推进。建立税费征收保障平台，财税收入质量进一步提高。

招商引资成效显著。坚持“走出去”和“请进来”相结合，深入开展主题招商活动，促成了一批项目签约落地。落实出口扶持政策，引导企业开拓新兴市场。实际利用外资增长15.6%，外贸出口增长13.5%。

(七)社会事业蓬勃发展，社会治理全面加强

就业再就业工作成绩显著。新增城镇实名制就业5万人，城镇登记失业率控制在2.5%以内，零就业家庭保持动态为零。社会保障体系不断完善，企业退休人员养老金、城镇医疗保险、城乡低保、医疗救助、最低工资标准等保障水平进一步提高。养老服务体系建设得到加强。

各项社会事业实现新发展。市一高中续建、九中改造、回族幼儿园翻建等工程全部竣工。调整市直学校布局，优化配置教育资源，促进了教育均衡发展。国家基本药物制度覆盖面不断扩大，基层医疗卫生机构乡村一体化工作扎实推进。创建国家历史文化名城，完成了历史文化名街评选申报。圆满完成了十二运赛事的承办任务。国防教育和国防动员建设进一步加强，创建全国文明城市工作步伐加快。国家安全、民族宗教、外事侨务、涉台事务、海关、检验检疫、质监、气象、防震减灾、人防、人口和计划生育、妇女儿童、物价、地方志、统计、档案、慈善等事业都取得了新的进步。

社会治理明显加强。整合基层信访维稳中心和公共服务中心，在全省率先设立乡镇街道社会管理综合服务中心。深入开展命案攻坚、打黑除恶等专项行动，健全社会治安防控体系，刑事案件发案率显著下降。完善稳定风险评估机制，集中化解信访积案，维护了社会和谐稳定。开展医疗纠纷调解工作，收到良好的社会效果。落实安全生产责任制，安全生产形势保持平稳。强化日常监管，提升了饮食用药安全保障水平。

(八)民主法制建设不断加强，政风建设水平显著提高

主动接受市人大及其常委会的法律监督和工作监督，认真执行市人大及其常委会的决议和决定，办理人大代表议案、建议249件。自觉接受市政协的民主监督，办理政协提案301件。认真听取各民主党派、工商联、无党派人士和人民团体的意见和建议。积极支持各人民团体依照各自章程开展工作。

认真落实关于改进工作作风、密切联系群众的各项规定，提高机关工作效能，政风建设显著加强。政府法制建设扎实推进，依法行政水平不断提升。公共行政服务审批程序进一步规范。行政监察、审计监督力度加大。政务公开向基层延伸。“六五”普法成效显著。推进党风廉政建设责任制全面落实，公共资源交易系统建设取得重大进展。

各位代表，保障和改善民生，是政府一切工作的出发点和落脚点。办好18项民生工程，解决涉及群众切身利益的问题，是新一届政府做出的庄严承诺。一年来，市政府坚持把改善民生作为头等大事来抓，组织各部门合力攻坚，确保18项民生工程按计划完成，用实际行动兑现了我们的诺言。

城市供水设施改造项目全面完成，供水质量明显提高。净水厂及输配水管线建设主体完工，今年6月可投入使用。新建农村安全水源30处，解决了5.3万人饮水安全问题。老城区农贸市场建设快速推进，三义市场、解放路市场正式营业;2处新建市场开工建设，今年投入运营。建设廉租房和公租房1300套，解决了部分低收入家庭居住问题。维修“弃管楼”202栋，改善了1万多户居民的居住环境。对23栋老旧楼房进行外墙保温改造，让1100多户居民住上了“暖房子”。更新环保型公交车100辆，方便了市民出行，提升了空气环境质量。公办幼儿园建设完成基础施工，今年将竣工。提高了新农合筹资和住院补偿标准，增强了农村居民健康保障能力。免费为64名先天性心脏病患儿进行了手术治疗。完成了市第四医院病房楼改造，改善了诊疗康复环境。新建村屯健身广场225个，为农村居民健身创造了条件。残疾人康复就业中心主体封顶，今年投入使用。新建农户储粮仓1.4万座，降低了储粮损耗。发放扶贫贴息贷款1.2亿元，年纯收入2500元以下的6.7万人实现脱贫。取缔高污染、高耗能企业69户，在全省率先实现了“一市一县一热源”，全市空气环境质量明显好转。新增高清监控探头1005个，建成了覆盖全市的视频监控系统，有力地震慑了犯罪，增强了人民群众的安全感。

上述成绩的取得，是全市人民团结奋斗的结果;是市人大法律监督、工作监督和市政协民主监督、参政议政的结果;是驻军部队，中央、省驻我市单位，各民主党派、人民团体、社会各界人士、外来投资者和建设者关心支持的结果。在此，我代表市政府，向支持参与辽阳经济社会建设的全市人民和各界人士表示衷心的感谢!

在总结成绩的同时，我们也清醒地看到经济社会发展中，还存在许多矛盾和问题。主要是：经济总量亟需做大，培育建设重大项目和产业集群的工作需要进一步加强;经济结构不尽合理，服务业占比较低，新兴产业发展步伐不快;新城新市镇发展不平衡，建设力度还需不断加大;资源环境约束加剧，节能减排和环境保护压力较大;政府职能转变还不到位，“四风”问题还不同程度地存在，社会治理和公共服务水平有待提高。对于这些问题，我们将采取有效措施，切实加以解决。

二、2014年工作安排

2014年是深入贯彻党的十八届三中全会精神、全面深化改革的起步之年，做好今年的工作意义重大。面对严峻复杂的经济形势，我们必须进一步增强进取意识、机遇意识、责任意识，以改革统揽发展全局，以改革开创工作新局面，努力推动全市经济社会发展实现新突破。

今年政府工作的总体思路是：高举中国特色社会主义伟大旗帜，深入贯彻党的十八大、十八届三中全会和中央经济工作会议精神，全面落实市委十一届六次全会的决策部署，紧紧把握稳中求进的工作总基调，把改革创新贯穿于经济社会发展各个领域各个环节，以科学发展为主题，以转变经济发展方式为主线，推进“三化”良性互动发展，为促进经济稳定发展、社会事业繁荣进步、民生持续改善而努力奋斗。

全市经济社会发展的主要预期目标是：地区生产总值增长9.5%左右，公共财政预算收入增长10%左右，固定资产投资增长20%左右，城镇居民人均可支配收入增长10%左右，农村居民人均纯收入增长11%左右。

为实现上述目标，我们要突出抓好以下八项工作。

(一)加强新城新市镇建设，加速沈辽一体化进程

提升环境品质，培育铝合金精深加工产业，实现河东新城开发建设的新突破。深入推进“一河两岸”景观带建设，实施新城整体亮化。完善基础设施，提高新城管理水平。取直新运大桥西侧道路，实现襄平大桥竣工通车。推进中轴线两侧商住开发，加快建设中南购物中心、红星美凯龙家居广场等商业设施。发展便民服务业，方便已入驻居民生活。推进铝合金精深加工产业基地建设，完善基础设施，加大招商引资力度，全力引进项目入驻基地。

其它新城新市镇要按照“三化”互动发展要求，统筹兼顾，因势利导，完善基础设施，做强主导产业，优化区域环境，促进产城融合。

推进沈辽一体化进程，增强区域发展优势。建成沈辽产业大道，加强与沈阳市在发展规划、产业布局、交通网络等方面的对接，促进优势互补。

(二)推动产业结构优化升级，提高新型工业化水平

推进工业经济平稳发展。健全工业经济运行监测平台，及时解决出现的问题。深入实施工业“五项工程”，提高工业经济核心竞争力。加大服务企业力度，优化企业投资和发展环境。营造全民创业氛围，激发民营经济发展活力。

推动产业结构优化升级。强化要素保障，力促200万吨铝板带箔项目全面开工建设，依托铝板带箔资源优势，积极研发新产品，引进配套项目，努力打造全国重要的铝合金精深加工基地。支持辽化公司调整产品结构，利用芳烃和烯烃产能优势，引进下游企业，增强就地加工转化能力，做大做强芳烃及精细化工产业集群。推进钢铁产业转型升级。抓好经济开发区建设，加快传统装备制造业转型升级步伐。

提升创新驱动能力。推进科技协同创新，建设研发、质监公共服务平台，支持企业实施技术改造，培育和引进研发团队，促进科技成果转化应用。发展信息产业，推动信息化与工业化深度融合。

强化生态环境治理，推进节能减排。深入实施“青山碧水蓝天”工程，推进矿山地质环境综合治理，治理新开河、运粮河和南沙河，全面完成年度淘汰落后产能任务，严控污染物排放，强化节能监管，严格环境执法，改善空气环境质量。

(三)构建新型农业经营体系，加快发展现代农业

创新农业经营机制，加速土地承包经营权规范有序流转。出台政策措施，建立健全服务体系，有序推进土地向专业大户、家庭农场、农民合作社和农业企业流转，发展多种形式的规模经营。

大力发展现代农业。编制完成全市现代农业发展规划。建设粮食高产示范区、现代农业示范区，提高农业综合生产能力。巩固水稻生产全程机械化成果，提高玉米生产全程机械化水平。大力推进农业产业化。做大做强“一县一业”。加强新型农村经济组织建设。健全农业技术推广服务体系。强化农产品质量安全监管，做好动物疫病防控工作。扩大农业保险规模，基本实现农业保险全覆盖。

发展生态友好型农业，改善农村生产生活环境。坚持耕地保护制度，统筹推进农田基础设施建设。实施灌区节水改造，加强防洪工程建设。推广农村新能源。实施农村危房改造，改善贫困农民居住条件。

加大开发式扶贫力度。开展产业项目进村入户行动，抓好扶贫培训，提高山区农民自我发展能力。

(四)推动服务业繁荣发展，增强城区创新发展能力

大力发展特色服务业。创建国家级生态旅游示范区，打造全国重要的皮草产销基地，加快建设温泉旅游和皮装裘皮集聚区，做大做强温泉冰雪、皮装裘皮购物两大特色旅游品牌。深度开发古城历史文化资源，促进文化与旅游有机融合。加速发展沟域旅游、乡村旅游。

提升传统服务业的规模和档次。推进白塔中央商贸区建设，启动建设站西商务区，拓展传统服务业发展空间。强化商业网点规划，培育餐饮服务业特色街区。引进新型业态改造传统商贸业。

积极发展生产性服务业。支持地方金融机构发展，积极引进外埠商业银行在我市设立分支机构。发展商贸物流业，加强物流园区建设，培育发展第三方物流企业，推动生产性服务业与制造业融合发展。

指导城区健全组织机构，完善配套措施，确保放权工作平稳过渡，增强城区创新发展能力。

(五)加强城市建设管理，努力营造宜居环境

加速推进老城区改建。加快重点地块房屋征收进度，改造城市棚户区、城中村和城边村。推进中心城区企业“退二进三”。实施繁荣路、振兴路两侧绿化改造，改善城市出入口形象。

完善城乡基础设施。大中修和改扩建县级以上公路180公里、农村公路140公里，完成村级道路建设200公里。启动城市公交枢纽站建设工程，建设交通信息指挥中心。完成净水厂及输配水管道建设。启动生活垃圾焚烧发电项目。加大市政道路维修改造力度，改善城市排水设施。实施“绿化亮化净化美化”提升工程，着力扩大绿地、水系、湿地等绿色生态空间比重，加速创建国家园林城市。加强电网建设，提高用电保障能力。

保持房地产业健康稳定发展。落实促进房地产业发展的政策措施，坚持项目开工并联审批制度，办好房交会，促进房地产投资持续稳步增长。

强化城市管理。推进城市管理精细化、智能化，提高管理质量和效率。深入开展侵占居民小区公共场地整治行动，完善小区设施，加强物业管理，改善人居环境。强化综合执法监管。大力整顿交通秩序。加强供暖管理，保障市民温暖过冬。

(六)深化改革扩大开放，充分激发经济发展活力

深入贯彻落实党的十八届三中全会精神，按照中央和省关于全面深化改革的战略重点、主攻方向、推进方式和时间表、路线图，积极研究和推进全市的改革发展，在重点领域和关键环节的改革上实现新突破。

加快转变政府职能，进一步简政放权，深化行政审批制度改革，加强和优化公共服务。积极稳妥推进行政管理体制改革，完成食品药品监管体制改革。深化市属国有企业改革，实现国有股彻底退出竞争性行业。积极推进集体企业改革，加大债权清收力度，盘活企业闲置资产。支持民营企业创新体制机制，加快上市进程。深化医药卫生体制改革，提高农村基本医疗卫生服务水平。

加大招商引资力度，提升对外开放水平。创新招商引资体制机制，拓展重点招商区域，优化投资环境，深入开展主题招商活动，提高招商引资的质量和水平。培育出口基地，积极开拓新兴市场，努力扩大出口规模。

(七)全面加强社会建设，切实增进民生福祉

以创业带动就业，以培训促进就业，积极创建国家级职业培训示范城市。扩大社保覆盖面，着力解决城乡各类特殊群体的社会保障问题。

办好人民满意的教育。加速发展学前教育，建成市直公办幼儿园。深化素质教育，规范中小学办学行为。巩固提高职业教育成果。实施市二高中、一中分校、六中等学校改造工程，改善办学条件。加强师德教育，强化行风监管。

提高公共卫生保障能力。启动居民健康档案建设工程，推进医疗卫生机构信息化进程。改扩建市第二医院、市第九医院、儿童医院、结核病医院综合病房楼，改善群众就医环境。遏制过度医疗行为，构建和谐医患关系。

大力繁荣文化事业。深入实施文化惠民工程，努力构建覆盖城乡、惠及全民的公共文化体系。加强历史文化遗产保护，弘扬古城文化，加速创建国家历史文化名城。广泛建立城乡体育指导站，就近指导广大群众科学健身。

加强和创新社会治理。深入开展平安创建活动，健全社会治安防控体系，依法严密防范和惩治各类违法犯罪活动，不断增强人民群众的安全感。深化矛盾纠纷排查，分类定性，统筹化解，有效解决各类社会矛盾。强化生产安全、消防安全、交通安全和食品药品安全监管，提高突发事件应急处置能力。促进民族团结进步，保持宗教和谐稳定。加强全民国防教育和国防动员建设，巩固扩大双拥共建成果。深入开展群众性精神文明创建活动，加速创建全国文明城市。

(八)加强政府自身建设，打造人民满意政府

坚持科学理政。主动接受市人大及其常委会的法律监督和工作监督，及时向市人大及其常委会报告工作，全面落实市人大及其常委会的决议、决定和审议意见。自觉接受市政协的民主监督，积极支持政协参政议政。充分听取政协以及各民主党派、工商联、无党派人士和人民团体的意见和建议。加强调查研究，及时解决群众反映强烈的问题。完善决策程序，狠抓决策落实，提高政府执行力。

推进依法行政。认真贯彻落实依法行政实施纲要，把依法行政贯穿于政府工作的全过程。深入开展“六五”普法教育，提高全社会法治化管理水平。认真落实党风廉政建设责任制，深化“五大系统”建设，规范公共资源交易行为。

切实改进政风。深入开展党的群众路线教育实践活动，强化对各项工作的专项整治，狠刹“四风”，高效务实，集中精力促发展，千方百计惠民生。

各位代表，民生连着民心。践行群众路线、打造人民满意政府，必须为民多办实事。今年，市政府要重点办好21项民生工程，努力解决人民群众最关心、最直接、最现实的利益问题。

1、改造城市供水设施35处，提高供水质量。

2、新建农村安全水源26处，解决4万人饮水安全问题。

3、建设4万亩鲜活农产品生产基地，在中心城区设立一批直销点，让市民吃到放心的农产品。

4、建设廉租房600套，解决城市部分低收入家庭居住问题。

5、对20个老旧小区进行综合改造，为小区居民营造舒适的居住环境。

6、维修“弃管楼”200栋，解决部分老旧楼房失管问题。

7、对40栋老旧楼房进行保温处理，让更多的居民住上“暖房子”。

8、建设中华大街襄平贸易城过街天桥，方便市民通行。

9、对20条城市支路和街巷道路实施维修改造，改善居民出行环境。

10、对20个小区内道路进行大修，方便居民出行。

11、购置新能源公交车121辆，改善市民乘车环境。

12、维修改造公厕66座，解决市民如厕难问题。

13、建设太子河生态公园，将“一河两岸”打造成市民休闲健身的理想场所。

14、对护城河中华大街至新运大街段、喇嘛园段实施综合改造，建成两处景观游园，为市民提供良好的休闲娱乐场所。

15、以绿化亮化净化美化为重点，完成131个行政村的环境整治，提升农村环境质量。

16、实施标准化教室建设，为8所中学配备“班班通”设备，更换标准化升降桌椅，改善教学条件。

17、实施图书馆二期工程，建设24小时自助图书馆，丰富市民业余文化生活。

18、对全市35岁以上的农村居民，实施以高血压综合防治为重点的健康管理，提高农村居民健康保障水平。

19、建设公益性公墓，降低群众安葬费用支出。

20、实施贫困单亲母亲关爱工程，帮助贫困单亲母亲解决居住、健康、子女就学等困难，让她们更加积极健康地生活。

21、实施贫困残疾人帮扶工程，帮扶贫困残疾人康复、就业，改善生活条件，提高生活质量。

我们要把群众的安危冷暖时刻放在心上，坚持不懈地增进民生福祉，不断提升广大群众的幸福感!

各位代表，新一年的征程已经开启，新跨越的目标催人奋进。让我们在市委的坚强领导下，开拓进取，奋力拼搏，为建设更加富裕、更加美丽、更加幸福的古城辽阳而努力奋斗!
